# Conceptualizing the Dynamics between Bicultural Identification and Personal Social Networks

**DOI:** 10.3389/fpsyg.2017.00469

**Published:** 2017-03-31

**Authors:** Lydia Repke, Verónica Benet-Martínez

**Affiliations:** ^1^Department of Political and Social Sciences, Universitat Pompeu FabraBarcelona Spain; ^2^ICREA (Catalan Institution for Research and Advanced Studies)Barcelona, Spain

**Keywords:** social networks, acculturation, immigration, cultural identification, complex contagions

## Abstract

An adequate understanding of the acculturation processes affecting immigrants and their descendants involves ascertaining the dynamic interplay between the way these individuals manage their multiple (and sometimes conflictual) cultural value systems and identifications and possible changes in their social networks. To fill this gap, the present research examines how key acculturation variables (e.g., strength of ethnic/host cultural identifications, bicultural identity integration or BII) relate to the composition and structure of bicultural individuals’ personal social networks. In Study 1, we relied on a generationally and culturally diverse community sample of 123 Latinos residing in the US. Participants nominated eight individuals (i.e., alters) from their habitual social networks and across two relational domains: friendships and colleagues. Results indicated that the interconnection of same ethnicity alters across different relationship domains is linked to cultural identifications, while the amount of coethnic and host individuals in the network is not. In particular, higher interconnection between Latino friends and colleagues was linked to lower levels of U.S. identification. Conversely, the interconnection of non-Latino friends and colleagues was associated with lower levels of Latino identification. This pattern of results suggests that the relational context for each type of cultural identification works in a subtractive and inverse manner. Further, time spent in the US was linked to both Latino and U.S. cultural identifications, but this relationship was moderated by the level of BII. Specifically, the association between time in the US and strength of both cultural identities was stronger for individuals reporting low levels of BII. Taking the findings from Study 1 as departure point, Study 2 used an agent-based model data simulation approach to explore the dynamic ways in which the content and the structure of an immigrant’s social network might matter over time in predicting three possible identity patterns: coexisting cultural identifications, conflicting cultural identifications, and a mixture of the two. These simulations allowed us to detect network constellations, which lead to identification or disidentification with both cultures. We showed that distinct patterns of social relations do not lead to identity outcomes in a deterministic fashion, but that often many different outcomes are probable.

## Introduction

Allport’s (1954) theory on intergroup contact states that, under certain conditions, contact between members of minority and majority groups will not only reduce prejudice and conflict, but will also improve interethnic attitudes (Pettigrew and Tropp, 2000; Binder et al., 2009). Nowadays, various scholars agree that one prerequisite for immigrants’ successful and peaceful integration into their host society is that they develop social networks which include host culture contacts in central positions, as these contacts provide access to critically important social and informational resources (Smith, 2013; Damstra and Tillie, 2016). These host nationals may improve the immigrant’s acculturation potential by helping with the acquisition of culturally appropriate skills and by providing exposure to new norms and value systems (Ward and Kennedy, 1993; Kim, 2001; Smith, 2005, 2013; Jasinskaja-Lahti et al., 2006). However, contact with coethnic individuals (living in the country of origin and in the country of destination) is beneficial as well. Coethnic friends and relatives living back home may give social support, safeguard the immigrant’s ethnic identity and skills, and even encourage adjustment to the new society (Lebon, 1983; Smith, 1999; Schultz, 2001). Similarly, coethnics in the country of destination may give important information and access to resources related to adapting to the host society (e.g., where and how to find a job), reducing the immigrant’s costs and risks in the country of settlement (Liu, 2013). Having said this, a social network comprised of too many coethnic individuals might be a burden to the immigrant’s acculturation potential, as the immigrant may feel pressured to hold on to habits or customs from the country of origin and may also lose an opportunity to learn and practice the host culture behaviors and norms (Luo and Wiseman, 2000). Ultimately, these processes may depend on the available social network opportunities, how much ethnic and host cultures objectively differ from each other (i.e., how much new cultural learning is called for), and whether the individual internalizes the differences as reflecting cultural conflict and incompatibility (Searle and Ward, 1990; Benet-Martínez and Haritatos, 2005).

Even though patterns such as low levels of identification with the host society and scarce friendships with host individuals are widely recognized in the literature, their interrelation is still open to question. Leszczensky (2013), for example, finds only a spurious relationship between degree of national identification and share of host national friends. Given the importance of social networks for integration and acculturation, it is surprising that hardly any study has examined how key acculturation variables (e.g., ethnic and host cultural identifications, bicultural identity integration, BII) relate to the composition (who is in the network) and structure (how are the network members connected) of immigrants’ personal social networks. Up to now, only a few sociological studies have attempted to do so, but did not include psychological measures (e.g., Lubbers et al., 2007; Vacca et al., 2016; but see also Mok et al., 2007). In particular, the relational perspective offered by the social network approach is suited perfectly for the acculturation and immigration context, as it captures intercultural contact in a way that goes beyond the commonly used self-reports. Most psychological research, including acculturation studies, focuses almost exclusively on individual-level characteristics (e.g., self-reported values and behaviors) in an effort to mirror what happens *inside* of people’s minds. But human behavior is also shaped by what happens *between* people’s minds. In this paper, we study how individuals’ cultural identities are influenced by their relational contacts, and the interactions that these contacts have *between* each other (Brown and Zagefka, 2011; Postmes et al., 2015).

We hypothesize that, in order for immigrants and their descendants to develop and strengthen their cultural identifications, repeated contact with culture-specific, attitude-relevant information (such as communication styles, cultural activities, gender roles, etc.) from individuals representing different roles is needed. The rationale behind this is the idea of complex contagions, which attests that certain social behaviors may only be changed after having had multiple contact with a variety of sources (e.g., as this adds credibility to the information received) (Centola and Macy, 2007; Centola, 2010). Behavioral changes then would be reflected in a change in cultural identification. More particularly, we argue that it is the interconnection of same ethnicity contacts belonging to different relationship domains (i.e., friendship versus work) that predicts the strength of individuals’ multiple cultural identifications.

In two separate studies, we explored possible relationships between key acculturation variables and personal social networks of immigrants and their descendants. In Study 1, we derived predictions for ethnic and host culture identifications from the idea of complex social contagion, and tested them using survey and network data collected from a community sample of 123 Latino-American biculturals residing in the US. In Study 2, using an agent-based model (ABM), we simulated data on the basis of the findings from Study 1 and explored whether and how the content and the structure of a bicultural individual’s social network matters over time in negotiating coexisting cultural identifications, conflicting cultural identifications, and a mixture of the two (e.g., being conflicting with regards to one life domain, but coexisting in another one).

We believe that our contribution to the study of multiple identities management in the acculturation context is twofold. From a scientific point of view, we will shed light on the unexplored possible interdependence between the micro-level represented by individuals’ self-reported acculturation processes (e.g., strength of cultural identifications and degree of conflict the individual feels between different cultural orientations) and the meso-level represented by these individuals’ habitual personal social networks. The fact that, in Study 2, we adopt a process-oriented rather than an outcome-oriented approach allows us to understand better how changes in the network may influence the identity negotiation process of immigrants and their descendants. From a societal point of view, our studies are informative in that they could be used to assist policy-makers involved with the integration of immigrants and other cultural minorities. In particular, the models examined in Study 2 could be used to identify social environments (i.e., specific network constellations) that are beneficial for fostering harmonious multicultural identities, and those that could lead to the development of risky patterns of cultural disidentification or radicalization. The paper is structured in the following way: First, we present our theoretical framework and our predictions. Then, we describe our two studies and their results. Finally, we summarize our main findings and offer some suggestions for future research in the discussion.

## Theoretical Framework and Predictions

### Acculturation, Cultural Identification, and BII

When moving to a new country, immigrants and their descendants often experience radical changes in their social and cultural contexts. The resulting acculturation processes may be described as psychological and behavioral changes that occur due to intercultural contact (Gibson, 2001; Sam and Berry, 2010). These processes oftentimes involve managing multiple, and sometimes conflictual, cultural value systems and identifications, and they also lead to changes in individuals’ social networks. These changes may include the creation of new relationships, the dissolution of old ones, or simply the diminishment or the consolidation of existing connections.

Generally, cultural identification can be understood as the sense of belonging to a cultural group (Nguyen and Benet-Martínez, 2007). In particular, long-term immigrants and their descendants may feel attached to not only one, but several cultures. As people who have been exposed to and who have internalized at least two sets of cultural meaning systems (e.g., beliefs, values, behaviors, languages), these individuals may be described as bicultural or multicultural (Hong et al., 2000; Nguyen and Benet-Martínez, 2007). Multicultural individuals have the capacity to acquire and use several cultural frames, even when these may be conflictual. Experimental research has shown that, depending on the available contextual cues, different cultural frames become salient, and that identification with a cultural group can shift accordingly (Hong et al., 2000, 2003; Verkuyten and Pouliasi, 2006; Verkuyten and Yildiz, 2007). This suggests that cultural identification is rather dynamic, and as such, neither primordial nor predefined, and thus it can undergo change (Lubbers et al., 2007).

Although it has been shown that acculturating individuals prefer the integration mode (i.e., being involved with both the ethnic and host cultures) (Benet-Martínez et al., 2002), these individuals may vary in how much they integrate their different cultural orientations and identities into a coherent sense of self (Huynh et al., 2011). Some biculturals may internalize cultural differences as reflecting conflict and incompatibility, while others may view their cultural orientations as compatible and even blendable. The construct of BII captures these differences and has become a central focus of empirical research on biculturalism (Benet-Martínez et al., 2002; Benet-Martínez and Haritatos, 2005). People high on BII view their two cultural identities as compatible and feel part of a combined (sometimes third) culture, whereas individuals low on BII consider their cultural identities as conflictual and dissociated from one another. The validity of BII as a psychologically meaningful construct has been well-established over the past decade, with research pointing to a wide variety of benefits associated with higher levels of integration (for reviews see Huynh et al., 2011; Benet-Martínez, in press).

### Personal Social Networks: Relational Domains and Social Contagion

According to the social network analysis framework, social networks consist of nodes and ties. Nodes are actors (e.g., individuals, groups, organizations), and their ties are connections (i.e., social relations) between them. While sociocentric network studies typically focus on complete networks, personal social network studies take the perspective of one particular actor. This focal node is the respondent in the study and is referred to as ego, which is why these studies are also called egocentric network studies. The members of ego’s network are called alters.

In Study 1, we were particularly interested in two relational domains that personal social networks commonly entail and that cross-cultural research often highlights (Pouliasi and Verkuyten, 2007): (1) close friends (excluding family members) and (2) classmates, co-workers or colleagues (who are not friends)^1^. Even though the interpretation of the term friend is culture and language specific (Scheuch, 1968; Fischer, 1982), people have more or less an understanding of what a friend is. Generally speaking, individuals tend to choose their friends freely from the social contexts available to them. They are not born into a circle of friends, like they are born into a family whose members are to a wide extent given. As such, people have some influence on the composition of their friendship network. Further, individuals might influence the structure of their friendship network by introducing friends from different areas of life to each other or by keeping them intentionally separate. Nevertheless, ego’s close friends tend to be engaged in each others’ social lives, whereas ego’s acquaintances are likely to not be involved with one another (Granovetter, 1983).

Colleagues, on the other hand, are often more given than selected freely. In some cases, people might have some influence on who becomes a colleague, but, normally, they cannot choose them as they wish. For instance, who becomes a colleague depends on who applies for a job, whether a particular candidate matches the job description, gets selected to fill in the position and, then, also accepts the offer. However, individuals may indirectly determine who their colleagues are by specifically deciding to work in an environment that is ethnic homogenous versus heterogeneous, or mainly coethnic versus non-coethnic. Yet, they are more restricted in choosing their colleagues than in selecting their friends. Further, people’s influence on how their colleagues are connected among each other might be quite limited as well, as the structure is often given by the company’s internal organization. In contrast, connections between friends and colleagues are usually not imposed by some third party and do not occur as naturally as maybe among friends, which leaves more freedom for ego to actively initiate relationships between alters of different relational domains (**Table 1**).

**Table 1:** Ego’s influence on network by relational domains.

	Ego’s influence on network		
	
	Within		Between
		
Alter relationship type	Composition	Structure	Interconnection
Friends	High	Medium	


			Very high
Colleagues	Low	Low	


Previous research has shown that friendships between immigrants and natives are positively linked to identification with host culture (e.g., Phinney et al., 2006; Leszczensky, 2013), and that friendships among coethnics are positively associated with higher levels of ethnic identification (e.g., Phinney et al., 2001; Ono, 2006). Yet, these studies did not actually measure social networks, but rather relied on self-reported number of friendships or frequency of contact (for exceptional examples see Lubbers et al., 2007; Mok et al., 2007). Social desirability and other types of biases might influence these responses. People may lie consciously about their social interactions with others, or may be influenced by memory biases and wishful thinking. In contrast, social network data does not rely on people’s self-assessment of their social lives, and instead maps onto actual contact between people. In this way, the network data collection mode is a more implicit and less obtrusive approach, and yields less danger of being actively manipulated by the respondent (Molina et al., 2014). Ergo, in the context of studying acculturation, the network approach more adequately grasps real-life situations of intercultural contact, while also measuring directly and more objectively with whom an individual interacts. Relying on real network data, we argue that the interconnection between friends and colleagues of the same ethnicity is a stronger predictor for cultural identification than the mere amount of alters belonging to a particular ethnic group. Our assumption is that receiving attitude-relevant information from individuals representing different roles (i.e., friend, colleague) strengthens the effect of attitude formation in the context of migration.

There are different theories about how behaviors spread via social contact in social networks. The most famous one is Granovetter’s (1973) seminal theory on the strength of weak ties (SWT). He defines the strength of a tie as a “combination of the amount of time, the emotional intensity, the intimacy […], and reciprocal services” (p. 1361). Usually, the concept of tie strength is measured by how well ego knows the network members or how close ego is to the alters (Marsden and Campbell, 1984). Duration of relationship, frequency of contact, and relationship categories are often used as proxies although, empirically, they are not necessarily correlated with tie strength. For example, family members do not need to have strong ties among each other, although in many cases immediate family members probably do.

The first premise of Granovetter’s theory states that the stronger the tie between two people A and B, the more likely it is that their social worlds overlap. So if A and B have a strong tie, and A and C have a strong tie, then, the likelihood for B and C to have at least a weak tie is increased (so-called transitivity). More concretely, one can expect that if A and B are good friends, and A and C are good friends, at some point in time, A will present B and C to each other (e.g., at a birthday party) and they might become also friends.

**FIGURE 1:**
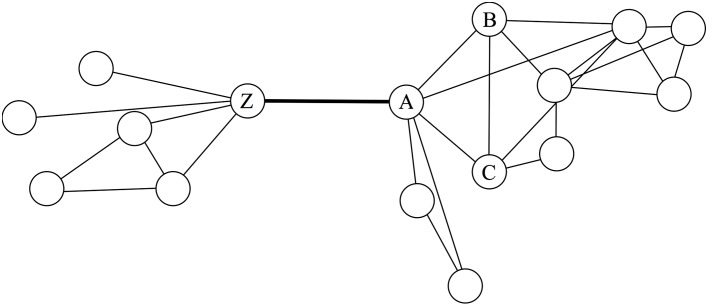
**Bridging tie**.

The second premise of his theory introduces the logic of bridging ties. This type of tie connects a person A to a person Z, who is not linked to A’s other contacts (**Figure 1**). This person Z may provide A with information that is different from what is already communicated in A’s other groups. This is because Z’s social world does not overlap with the social worlds of A’s other contacts, and, hence, is likely to be a distinct social environment with access to different information. In this sense, a bridging tie is seen as a “potential source of novel information” (Borgatti and Halgin, 2011, p. 1171). In an egocentric or personal social network that is reflected by having more separate groups (also referred to as structural holes; Burt, 1992) that would lead ego to possibly get more non-redundant, novel information at any given time (e.g., on the availability of a job offer).

In conclusion, classical network theory argues that less connected networks with many weak ties diffuse novel information, such as behavioral norms and values, faster and more effectively than networks with highly clustered ties. In this view, spread of behavior is understood as a simple contagion via social contact in the network. For example, simple contact with information relating to a score on a volleyball match or the time of a concert might be enough to inform an individual. In this regard, contact with one source is sufficient to change the behavior of one person. Hence, an immigrant’s personal social network with many weak ties would facilitate the efficient and fast spreading of culture-related behaviors and norms.

However, this may not always be the case and may depend on what it is that is being diffused. Especially for “costly, risky, or controversial” behaviors “independent affirmation or reinforcement from multiple sources” might be required (Centola and Macy, 2007, p. 703). The contagion then is not simple anymore but complex, because the individual needs to have contact to at least two different sources before credibility is assigned to the received information and a change in behavior is initiated. Especially in the acculturation context, we argue that contact with a single host culture individual is not enough for an immigrant to change host cultural identification. Instead, the immigrant may need repeated contact to several host nationals before a change in cultural identification may be activated. Similarly, a single contact to only one coethnic individual may not be enough to trigger such a change either, but repeated contacted to different coethnic individuals might be. Receiving the same information through repeated contact with different people is more likely in highly clustered networks. Thus, an immigrant’s personal social network with many redundant (i.e., strong) ties fulfills the structural conditions to provide the social affirmation and reinforcement mechanisms that are necessary for adapting a change in cultural identification.

The underlying network dynamic of social contagion is influence, which refers to the fact that individuals change their attitudes and behaviors in reaction to their network members. The complementing network dynamic is selection. It describes the process in which people choose their network members and is usually based on the principle of similarity. Both processes may lead to the same result, namely homophily of network members, and are usually interwoven (Veenstra et al., 2013). In this paper, we do not try to empirically cut this Gordian knot as the data reported in Study 1 is cross-sectional and Study 2 follows directly from its results, although we acknowledge the complexity and endogeneity that the relationship of cultural identification and social networks contains. We rather focus on one possible network dynamic (i.e., influence in form of complex contagions) for the purpose of theory-building.

Drawing on the literature of complex contagions, we argue that, similarly to adopting costly and risky behaviors, cultural identifications are not altered easily though they are dynamic in nature. They may change slowly over time instead of changing dramatically because of one simple contact with a certain culture. Thus, we hypothesize that when immigrants and their descendants develop and negotiate their cultural identifications, they may adopt changes in their identifications as a result of receiving repeatedly culture-relevant information from multiple network members. While, on the one hand, these different network members need to be interconnected to make social affirmation and reinforcement more likely, on the other hand, these network members need to be from different relational domains to enhance the credibility of the information. For this reason, we expect that the interconnection of same ethnicity alters from different relationship domains provides immigrants and their descendants with repeated information from different sources that may alter their cultural identifications in the long-run. Therefore, our main hypothesis is that the interconnection of same ethnicity alters across relational domains is a stronger predictor for cultural identification than the mere amount of alters belonging to a particular ethnic group.

### The Current Research

In the present work, we explored potential relationships between key acculturation variables (i.e., time in the US, ethnic and host cultural identifications, BII), and the content and the structure of personal social networks of immigrants. To do so, we conducted two studies. Using a cross-sectional, correlational design, Study 1 examines survey and personal social network data from 123 Latinos living in the US. The egocentric network data included eight alters: four friends and four colleagues (e.g., classmates, co-workers), thus tapping into two key relational domains to test the following three hypotheses:

H1: The interconnection between friends and colleagues of the same ethnicity is a better predictor for cultural identification than the size of the corresponding ethnic group.
H2: U.S. identification is positively associated with the interconnection of European-American friends and European-American colleagues.
H3: Latino identification is positively associated with the interconnection of Latino friends and Latino colleagues.

The second study builds on the findings of Study 1. Utilizing an ABM data simulation approach, we explore the dynamic ways in which network composition and structure may matter over time in predicting intrapersonal identification change.

## Study 1

In Study 1, Latino immigrants and their descendants living in the US were asked to complete a questionnaire about their cultural identifications, their bicultural experiences and their personal social networks.

### Method

#### Participants

We relied on a community sample consisting of 123 Latino-American biculturals (41 males, 81 females, 1 transgender), aged 16–65 years (*M* = 28.5, *SD* = 9.4; 70.6% with college education or higher), who voluntarily participated in this study. All participants were first- to fifth-generation immigrants living in the US, out of which: 28.5% were first generation, 8.1% 1.5 generation (migration to the US before the age of 16), 25.2% second generation (born in the US, parents born outside), 14.6% 2.5 generation (born in the US, one parent born in the US, the other parent born outside), 15.5% third generation (parents born in the US), 0.8% 3.5 generation (one pair of grandparents born outside of the US), 3.3% fourth generation (grandparents born in the US), and 4.1% were fifth generation (great-grandparents born in the US). Participants born abroad came mainly from Mexico (53.3%) or El Salvador (20.0%) and had spent on average 11.3 years (*SD* = 8.0) in the US. Other countries of origin include Brazil (*n* = 1), Chile (*n* = 1), Costa Rica (*n* = 2), Cuba (*n* = 1), Ecuador (*n* = 1), Guatemala (*n* = 1), Nicaragua (*n* = 2), and Spain (*n* = 1). The parents of the participants who were born in the US came mainly from the US (mothers: 50%; fathers: 47.4%) or Mexico (mothers: 26.9%; fathers: 25.6%). The other mothers were born in Colombia (*n* = 3), Cuba (*n* = 1), El Salvador (*n* = 6), Guatemala (*n* = 2), Nicaragua (*n* = 1), Peru (*n* = 1), or Puerto Rico (*n* = 3), and the rest of fathers was born in Colombia (*n* = 1), Cuba (*n* = 1), Czechoslovakia (*n* = 1), El Salvador (*n* = 6), Guatemala (*n* = 2), Italy (*n* = 1), Korea (*n* = 1), or Puerto Rico (*n* = 3).

#### Procedure

Participants for Study 1 were recruited at a public “Cinco de Mayo” street festival taking place in downtown San Francisco in 1997. Individuals present at the festival area were either politely approached by the experimenter and her assistant (all of whom were both Latino and Spanish-English bilinguals) or voluntarily came to a booth where a table sign saying “Are you bicultural? Contribute to science and our better understanding of the Latino experience” was displayed. All subjects completed the paper-and-pencil survey privately and anonymously and gave written informed consent. The survey requested basic demographic information and included the measures described below. No questions about immigration legal status were asked in the survey.

The study was carried out following ethical guidelines and in accordance to UC Berkeley’s Committee for the Protection of Human Subjects (Part VI, B, 3, a, i), which approved the study. The study was completely anonymous, did not include questions of sensitive nature, did not involve deception, and did not pose any anticipated risks to the participants.

#### Instruments

Participants completed a questionnaire that was made available in both Spanish and English, designed to measure the following variables:

##### Acculturation-related measures

###### Cultural identifications

Ethnic and host cultural identifications were measured with two separate items that read “I feel North-American (U.S.)” (U.S. identification) and “I feel Latino/Hispanic” (Latino identification). The response scale ranged from 1 (*strongly disagree*) to 6 (*strongly agree*). The average levels of identification were 3.9 (*SD* = 1.7) for U.S. identification and 5.3 (*SD* = 1.0) for Latino identification. The correlation between the two identification scales was *r* = -0.31 suggesting that, at least for this sample, identification as an American and as a Latino was experienced as moderately oppositional.

###### Bicultural identity integration

Bicultural identity integration was measured with four force-choice items each tapping high versus low BII (e.g., “I combine both cultures” versus “I keep both cultures separate,” “I don’t feel caught between the two cultures” versus “I feel caught between two cultures”). For each answer option that corresponded to high BII we gave one point, zero if otherwise. The final total score ranged from 0 to 4 (ordinal alpha = 0.68).^2^ Given the shortness of this scale (four non-redundant items tapping different facets of identity integration), this alpha is satisfactory. Overall, participants reported a BII mean level of 2.6 (*SD* = 1.2).

###### Time in the US

This variable reflects the approximate total amount of years the respondent had lived in the US at the time of the survey. Among second generation participants this variable might very closely reflect the respondent’s age minus the time spent outside the US.

##### Network-related measures

Participants were first asked to list their four closest friends in California, with whom they had interacted with as personal friends throughout the last year and who were not family members. Second, they named four classmates, co-workers or colleagues in California with whom they had interacted with the most during the last year and who were different from their friends. Participants wrote the initials of the nominated individuals (i.e., alters) in eight circles and were then given the instruction to draw lines among all the individuals who had a relationship (described as having frequent interactions or being friends themselves). As a last step, respondents coded the ethnicity of each alter using the following categories: Latino/Hispanic, Asian, African-American, European/Anglo-American, and other (please specify). From this data, we constructed two variables measuring the networks’ composition (who is in the network) and two variables measuring its structure (how are the network members connected).

###### Group size of Latinos

Group size of Latinos, as a compositional measure, is the absolute count of Latino alters in the network. With a network size of eight alters, the variable may take values between 0 and 8. Overall, participants listed 4.1 (*SD* = 2.3) Latino alters.

###### Group size of European-Americans

Likewise, group size of European-Americans refers to the absolute count of alters classified as European/Anglo-American and may range from 0 to 8. Overall, participants listed 2.2 (*SD* = 1.9) European-American alters.

###### Interconnection of Latino friends and colleagues

This structural variable is an indicator for how well Latino friends and Latino colleagues are on average connected to each other weighted in accordance to their group size (variable referred to as inter-class tie weight in Brandes et al., 2008). It is a normalized measure that is based on the idea of the average number of neighbors between two groups. Overall, the weight of how well Latino friends F and Latino colleagues C are connected to each other was 0.4 (*SD* = 0.6; *MAX* = 3.2). The weight is calculated as:

$$\omega (F,\;C)=\frac{e(F,\;C)}{\sqrt{\left|F|\cdot |C\right|}}$$

###### Interconnection of non-Latino friends and colleagues

In a like manner, this variable expresses how well non-Latino friends and non-Latino colleagues are connected to each other. Overall, participants’ two groups had an interconnection weight of 0.4 (*SD* = 0.7; *MAX* = 3.5).

Correlations for all measures are provided in **Table 2**.

**Table 2:** Correlation matrix for main variables.

	1	2	3	4	5	6	7	8
***Identification***								
1 Latino	-							
2 U.S.	-0.31***	-						
***Acculturation-related***								
3 Time in the US	-0.24**	0.37***	-					
4 BII	-0.14	0.25**	0.18†	-				
***Group size***								
5 Latinos	0.21*	-0.19*	-0.37***	-0.17†	-			
6 European-Americans	-0.16†	0.17†	0.29***	0.03	-0.77***	-		
***Interconnection***								
7 Latino F/C	0.17†	-0.27**	-0.25**	-0.09	0.52***	-0.33^∗∗∗^	-	
8 Non-Latino F/C	-0.21*	0.09	0.08	-0.00	-0.44***	0.23^∗∗^	-0.13	-


*1 Latino identification; 2 U.S. identification; 3 time in the US; 4 BII; 5 group size of Latinos; 6 group size of European-Americans; 7 interconnection of Latino friends and colleagues; 8 interconnection of non-Latino friends and colleagues. ^∗^*p* < 0.05. ^∗∗^*p* < 0.01. ^∗∗∗^*p* < 0.001.*

### Results

Our main hypothesis was that the interconnection of same ethnicity alters across relational domains would be positively linked to cultural identification. To test this, we ran two separate hierarchical multiple regressions, one set using U.S. identification as a dependent variable and another set using Latino identification as a dependent variable. We calculated three models for each regression. The first model included the acculturation-related variables time in the US and BII as predictors, and their interaction to test for a possible moderation effect of BII. We replaced the five missing cases in the variable time in the US with the overall sample mean to not lose valuable network data. BII had nine missing cases, but as it was operationalized as a composite score, we did not replace them. The reason for this is that for composite scores a big variety of replacement strategies exist, and the choice of one runs the risk of being biased in favor of the researcher’s interest. However, this decision led to the reduction of our sample size to 114. In the second model, we added the predictor variables group size of European-Americans for predicting U.S. identification and group size of Latinos when predicting Latino identification. For reasons of multicollinearity, we included only one group size variable at a time. In the final model, both interconnection variables were added.

**FIGURE 2:**
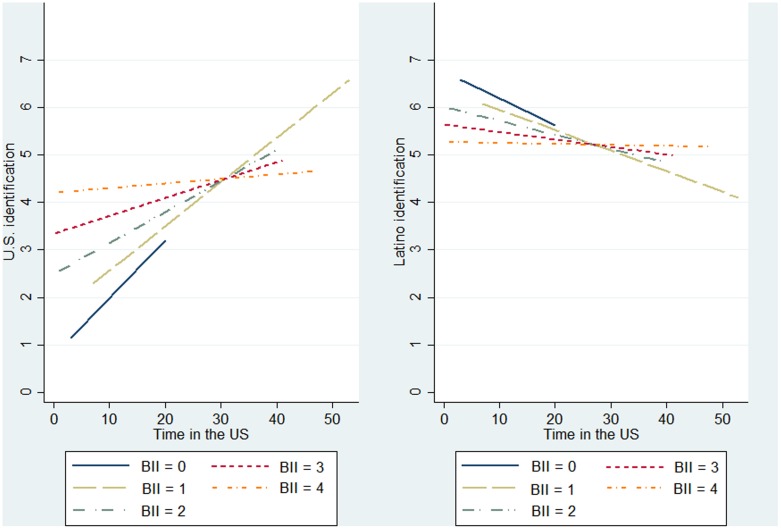
**Interaction term**.

The results for U.S. identification are shown in **Table 3**. Because of our small sample size, which makes the detection of significant effects difficult, and the fact that social network variables generally tend to show great variation (Brandes et al., 2008), we treat *p* values below 0.10 as significant (for similar procedure see Mok et al., 2007). Throughout all the models the acculturation-related variables were significant, indicating a strong positive association between U.S. identification with both time in the US and BII. Interestingly, this relationship was stronger for individuals scoring low on BII and lower for individuals scoring high on BII (see left side of **Figure 2**). In line with our expectations, we did not find any effect for group size, but an effect for one of the interconnection variables (H1). However, Hypothesis 2 (a positive link between the interconnection of non-Latino alters across relational domains and U.S. identification) was not supported. Instead, to our surprise, we found a negative link between U.S. identification and the interconnection of Latino friends and colleagues (β = -0.18; *p* = 0.043). Change in *R*^2^ between the models was not significant, but followed the trend of Hypothesis 1 that network structure is a better predictor than pure network content (from Model 1 to Model 2: Δ*R*^2^ = 0.01; *p* = 0.178; from Model 2 to Model 3: Δ*R*^2^ = 0.03; *p* = 0.124; from Model 1 to Model 3: Δ*R*^2^ = 0.04; *p* = 0.112). According to Akaike’s Information Criterion (AIC; DeLeeuw, 1992), in which lower values indicate a better fit, Model 3 (AIC = 417.455) fitted the data best (Model 1: AIC = 417.814; Model 2: AIC = 417.909).

**Table 3:** Regression results for U.S. identification.

	U.S. identification		
	
Predictor	1	2	3
1. Acculturation-related			
Time in the US	0.33***	0.29**	0.26**
BII	0.19*	0.19*	0.18
Time x BII	-0.23*	-0.24**	-0.22*
2. Group size			
European-Americans		0.12	0.07
3. Interconnection			
Latino F/C			-0.18*
Non-Latino F/C			0.02
*R*^2^	0.24	0.25	0.28
AIC	417.81	417.91	417.46


**N* = 114. Reported model coefficients are standardized betas. F/C stands for the interconnection of friends and colleagues. ^∗^*p* < 0.05. ^∗∗^*p* < 0.01. ^∗∗∗^*p* < 0.001.*

**Table 4** reports the regression results for Latino identification. Similar to the findings above, time in the U.S. and the interaction term were significant predictors, but at lower levels. The negative association between Latino identification and time in the U.S. was stronger for low BIIs (see right side of **Figure 2**). Again, one of the interconnection variables had a greater effect on identification than group size of Latinos when all network variables were included in the model (H1). We found a weak negative link between Latino identification and the interconnection of non-Latino friends and non-Latino colleagues (β = -0.18; *p* = 0.068) opposed to the hypothesized positive effect of the interconnection variable of Latino alters across relational domains (H3). In general, our models explained more variation in identification with the host culture than with the ethnic culture. Change in *R*^2^ was only marginally significant from Model 1 to Model 2 (Δ*R*^2^ = 0.02; *p* = 0.087) and from Model 1 to Model 3 (Δ*R*^2^ = 0.05; *p* = 0.094), but not from Model 2 to Model 3 (Δ*R*^2^ = 0.03; *p* = 0.176). Model 2 seemed to fit the data best (AIC = 320.277).

**Table 4:** Regression results for Latino identification.

	Latino identification		
	
Predictor	1	2	3
1. Acculturation-related			
Time in the US	-0.24*	-0.18†	-0.19†
BII	-0.10	-0.08	-0.09
Time x BII	0.19*	0.18*	0.17†
2. Group size			
Latinos		0.17†	0.05
3. Interconnection			
Latino F/C			0.06
Non-Latino F/C			-0.18†
*R*^2^	0.12	0.14	0.17
AIC	321.35	320.28	320.58


**N* = 114. Reported model coefficients are standardized betas. F/C stands for the interconnection of friends and colleagues. ^†^*p* < 0.10. ^∗^*p* < 0.05.*

### Discussion

Though the results of Study 1 were not very strong, they show that situating and investigating bicultural individuals in their social contexts may be a fruitful approach for understanding the dynamic process of cultural identification. The results suggest that structural aspects of the social context predict patterns of cultural identification better than pure compositional aspects. Specifically, the interconnection between Latino friends and colleagues was linked to lower levels of U.S. identification, while the interconnection of non-Latino friends and colleagues was associated (although more weakly) with lower levels of Latino identification. This culturally inverse pattern of results seems to indicate that, at least for this sample, the social context facilitated cultural identification with the group of interaction by suppressing or lowering the identification with the other culture. Overall, the results suggest a sense of tension or opposition between identifying as an American and as a Latino, leading to the conclusion that those two cultural identifications may be subtractive or oppositional. In particular, external contextual pressures, such as the 1994 California Proposition 187 which prohibited illegal immigrants from using certain public services (e.g., non-emergency health care, public education), might have signaled to the Latino-American biculturals of our study that they cannot be both. Moreover, some Latino groups in the US are highly stigmatized, which may also add to this competing pattern found for both individuals’ self-reported cultural identifications and the structure of the social networks that support these identities.

One reason for why our models explained host cultural identification better than ethnic identification might be that Latino identification is less malleable and strongly influenced by variables such as family and child socialization. Bicultural individuals might develop a strong sense of ethnic identity already in the family context and then later add a sense of belonging to the larger host culture. This feeling of belonging to the host society is probably more influenced by what happens and by what immigrants and their descendants do outside the family context, thus encompassing a wider scope of experiences.

Time in the US was associated to both cultural identifications, in that time spent in the US was linked to higher levels of U.S. identification and lower levels of Latino identification. This indicates that as the amount of exposure to and engagement with U.S. culture increases with time, Latino and U.S. cultural identifications become subtractive (for subtractive pattern see de la Sablonnière et al., 2016). Certainly, this pattern seems to be at odds with a bidimensional, two-directional, multidomain definition of acculturation (see Ryder et al., 2000; Flannery et al., 2001; Schwartz et al., 2010). However, an interesting feature of these results is that the subtractive pattern is especially strong among Latinos scoring low on BII, supporting the notion that BII taps into perceptions of cultural incompatibility and conflict.

As the signs in Study 1 for the hypothesized associations between cultural identifications and the interconnection variables (H2 and H3) were different than expected, in the next study we used simulations and an agent-based modeling approach to explore in a dynamic way some of the (static) patterns examined in Study 1. The design of the second study allows us to predict different patterns of intrapersonal change in cultural identification over time based on the composition and structure of this individual’s personal network. This more dynamic approach allows us not only to model the effects of social cues and social contexts (i.e., social network composition) on cultural identifications (Hong et al., 2000; Verkuyten and Pouliasi, 2006), but also to model the effects of structural aspects of the social context.

## Study 2

Study 2 served two main purposes: (1) to illustrate some of the static results reported in Study 1 in a dynamic way, by modeling intrapersonal change, thus tapping into the dynamic nature of cultural identification; and (2) to explore other possible multiple identities negotiation and management mechanisms. We designed an ABM and simulated data to demonstrate how an immigrant’s identification with host and ethnic cultures may change depending on the composition and structure of this individual’s personal network. This model may be useful in understanding complex identification outcomes evoked by simple mechanisms based on the principle of influence within networks. It further demonstrates why it might be difficult to detect consistent and strong network effects with regression analysis utilizing cross-sectional data. Moreover, this model may provide a promising starting point for informing the study of how multiple cultural identities and inter-group relations dynamically interact, and how these processes might lead to the emergence of particular identity structures such as hyphenated identities or identification with a third culture, and perhaps even the development of extreme patterns of cultural identification resulting from the disidentification with either of the other two or even both cultures. Our model allows us to explore the negotiation processes of coexisting identities, conflicting identities, and a mixture of the two. Because most psychologists are unfamiliar with ABM techniques, in the next section, we will briefly describe our model following the standard ODD protocol (Overview, Design concepts and Details) that ensures an easy understanding of the model (Grimm et al., 2006, 2010).

### Method

#### Entities, State Variables, and Scales

When developing our ABM in the NetLogo software (Wilensky, 1999), we tried to stay as close as possible to the operationalization of the variables used in the questionnaire of Study 1. As a consequence, we will describe the model specifically for the context of Latino-American biculturals, though it could be applied to any two cultures. The model consists of two types of entities: one ego and eight alters. Ego is characterized by *identification* with Latino (ethnic) culture and by identification with U.S. (host) culture, each possibly ranging from 1 to 6. Alters are characterized by their state variables *ethnicity* (either Latino or European-American) and *degree centrality* (here the amount of ties with same ethnicity alters, ranging from 0 to 7).^3^ The ties among the eight alters are undirected (meaning the alters have symmetric, reciprocal relationships that is, e.g., alters view each other as friends) and distributed randomly, ranging from 0 (alters completely disconnected) to 28 (alters completely connected). Throughout the simulation ego has 50 social interactions, always with one alter at a time. Each social interaction happens at a different point in time and may lead to a change in cultural identification. In that sense, time proceeds in discrete steps, and the length of each time step is not specified further. Composition and structure of the network are held constant through time.

#### Process Overview and Scheduling

Our model includes only one process: change of ego’s cultural identifications. Ego’s change in identification with Latino and U.S. cultures is traced throughout 50 time steps. At each time step, ego interacts randomly with one of the eight alters and changes level of identification with either one of the cultures, both, or none depending on the implemented rule. In total, we modeled three different mechanisms or rules: positive effect, negative effect and mixed effects (see **Table 5** for a summary).

**Table 5:** Formal comparison of effects.

	Effect		
	
	Positive	Negative	Mixed
Description	Interaction with alter *increases* cultural identification with the *same* culture (=culture of alter)	Interaction with alter *decreases* cultural identification with the *other* culture (≠ culture of alter)	Interaction with alter *increases* cultural identification with the *same* culture (=culture of alter) and *decreases* cultural identification with the *other* culture (≠ culture of alter)
Formalized description	Interaction with alter a increases identification with culture A. Interaction with alter b increases identification with culture B_._	Interaction with alter a decreases identification with culture B_._ Interaction with alter b decreases identification with culture A.	Interaction with alter a increases identification with culture A and decreases identification with culture B_._ Interaction with alter b increases identification with culture B and decreases identification with A.
Equation	*ID*_A_ + 0.1 + *d*_a_ ⋅ 0.1	*ID*_B_ - (0.1 + *d*_a_ ⋅ 0.1)	*ID*_A_ + α(0.1 + *d*_a_ ⋅ 0.1)
			*D*_B_ - (1 - α)(0.1 + *d*_a_ ⋅ 0.1)
	*ID*_B_ + 0.1 + *d*_b_ ⋅ 0.1	*ID*_A_ - (0.1 + *d*_b_ ⋅ 0.1)	*ID*_B_ + α(0.1 + *d*_b_ ⋅ 0.1)
			*ID*_A_ - (1 - α)(0.1 + *d*_b_ ⋅ 0.1)


**ID_A_* refers to ego’s initial identification with culture A and *ID_B_* to the initial identification with culture B. D is the degree centrality of the alter ego is interacting with and refers to the number of ties this alter has with other same ethnicity alters. α determines how strong positive and negative effects are.*

First, the *positive effect* describes a mechanism similar to the one we had originally hypothesized in Study 1, namely, that a social interaction with an alter of a particular culture will increase identification with that same culture. Similarly, if ego interacts with an alter of another culture, identification with this new culture increases. As a result, social interaction will always lead to an increase in identification with the culture of the alter ego is interacting with. In this sense, both cultural identifications coexist and are independent from each other. More concretely, whenever ego interacts with a Latino alter a, ego’s Latino identification increases by 0.1 + *d*_a_ ⋅ 0.1, where *d*_a_ (degree centrality) is the number of ties that alter a has with other Latino alters. Likewise, whenever ego interacts with a European-American alter b, ego’s U.S. identification increases by 0.1 + *d*_b_ ⋅ 0.1, where *d*_b_ (degree centrality) is the number of ties that alter b has with other European-American alters. Basically, at each social interaction one of the cultural identifications is increased by at least 0.1. We chose 0.1 as it is a basic mathematical unit of change between 0 and 1. The increase in identification is greater than 0.1 when the alter of an interaction has at least one tie to another alter of the same ethnicity. As we wanted to model cultural identification change over time, and, thereby, avoid reaching complete identification too fast (= 6, identification is measured from 1 to 6), we multiplied the degree centrality with 0.1 as a basic unit of change. We used degree centrality to model the idea that the relationships among alters or their social interactions matter for the identification of ego. The more same ethnicity ties an alter has, the greater is the influence on ego’s identification. We thus do not only examine the effects of the social context on cultural identification, but also its structural aspects.

Second, the *negative effect* is based on the actual finding from Study 1 showing that cultural identification with a particular culture decreases when an individual interacts with somebody of another culture. Ergo, social interaction always decreases identification with the culture ego is not interacting with. In that way, engagements with each culture coexist but are not independent from each other. As a consequence, interaction with one culture always leads to a reduction (i.e., suppression or lowering) of identification with the other culture. Precisely, this means that whenever ego interacts with a Latino alter a, ego’s U.S. identification decreases by 0.1 + *d*_a_ ⋅ 0.1. Similarly, whenever ego interacts with a European-American alter b, ego’s Latino identification decreases by 0.1 + *d*_b_ ⋅ 0.1. Again, a social interaction has more impact on the change of cultural identification when the alter of the interaction is better connected to other same ethnicity alters.

Third, the *mixed effects* version of the model is a combination of the former two mechanisms. At each social interaction, positive and negative effects take place simultaneously. In practical terms, this could be when both cultures are seen as coexisting with regards to one life domain (e.g., work values), but as conflicting with regards to a second one (e.g., gender roles). Another addition to the former two versions of the model is the variable α, which regulates the influence of the two effects. This variable ranges from 0 (negative effect is present, but positive effect is absent) to 1 (positive effect is present, but negative effect is absent). Only when α is equal to 0.5 both effects have the same influence on identification. For all other values, either the negative or the positive effect is stronger. Thus, whenever ego interacts with a Latino alter a, ego’s Latino identification increases by α(0.1 + *d*_a_ ⋅ 0.1) and ego’s U.S. identification decreases by (1 - α)(0.1 + *d*_a_ ⋅ 0.1). Likewise, whenever ego interacts with a European-American alter b, ego’s U.S. identification increases by α(0.1 + *d*_b_ ⋅ 0.1) and ego’s Latino identification decreases by (1 - α)(0.1 + *d*_b_ ⋅ 0.1). In all three versions of the model, ego may change both identities up to a maximum value of 6 and down to a minimum value of 1.

#### Initialization and Simulations

Before simulations began, ethnicity was assigned randomly to the eight alters. So was the distribution of their ties. At the start of each simulation, ego was set up to have moderate identification of 3.5 with Latino and US-American cultures (midpoint of the scales). Each simulation ended after 50 time steps and provided two outcomes: one value for ego’s Latino identification and one value for ego’s U.S. identification (later referred to as outcome identification). We simulated data by systematically varying the ratio between Latino and European-American alters (i.e., 0:8, 1:7, …, 8:0), and the amount of alter ties in steps of four (i.e., 0, 4, …, 28), resulting in 72 different combinations each for the positive and for the negative effect. Then, we tried the same combinations with the mixed effects model for α values ranging from 0 to 1 in steps of 0.1. Hence, we had 792 combinations for the third model. As running a model with certain initial values once may show only one possible development and result in only one outcome out of many (Bijak et al., 2013), we ran the simulation of each combination of starting values 50 times. Taking all three models together, we ended up with 46,800 values each for Latino identification and for U.S. identification (9_ethnicity ratio_ ⋅ 8_alter ties_ ⋅ (1_positive_ + 1_negative_ + 11_α_) ⋅ 50_repititions_).

### Selected Results

We computed means and their standard deviations for both outcome identifications and for each combination of starting values considering all 50 repetitions. **Tables 6**, **8** summarize the means of each of the two outcome identifications for positive, negative and mixed effects, respectively, and **Tables 7**, **9** their standard deviations. In the orange graphs, each of the nine orange lines represents a distinct composition of the network (ranging from dark orange with no Latinos and eight European-Americans to light orange with eight Latinos and no European-Americans). The *x*-axis gives information on the structure of the network (i.e., amount of alter ties), and the *y*-axis holds the mean of the outcome identification or its standard deviation. In the purple graphs, each of the eight purple lines represents a different network structure (i.e., amount of alter ties; the darker the line, the less alter ties), while the *x*-axis captures the composition of the network (i.e., amount of Latino alters). Again, the *y*-axis shows the mean or the standard deviation of the outcome identification given a specific network constellation (i.e., network composition and structure).

**Table 6:** Mean of identification outcome for negative and positive effects.

	Negative effect α = 0	Positive effect α = 1
Latino ID		
	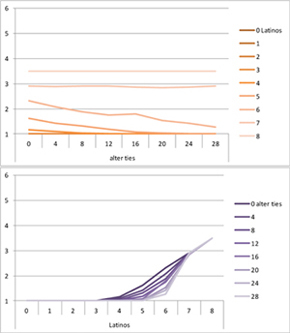	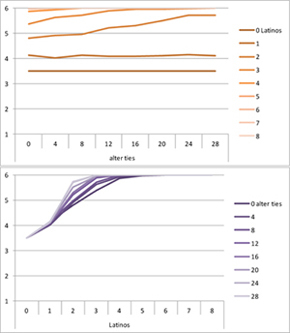

US ID		
	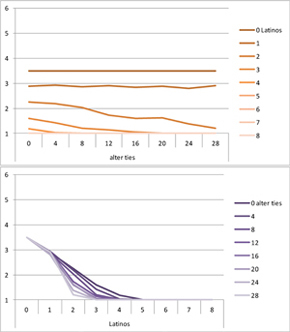	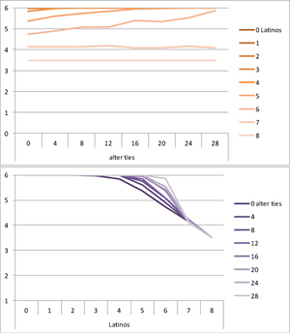
		


*Measurement points are shown as lines for visual simplicity. Some of the lines may be difficult to see as they overlap.*

**Table 7:** Standard deviations of means for negative and positive effects.

	Negative Effect α = 0	Positive Effect α = 1
Latino ID		
	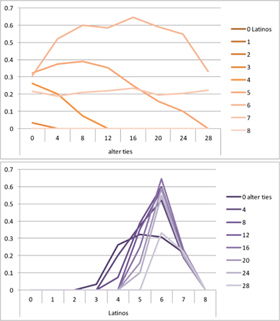	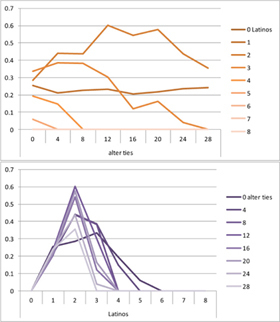

US ID		
	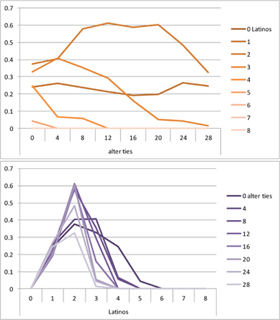	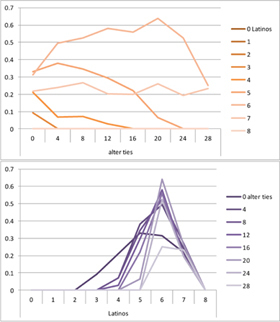


*Measurement points are shown as lines for visual simplicity. Some of the lines may be difficult to see as they overlap.*

To give some examples, in the mean plot of the negative effect model of Latino identification (upper left graph in **Table 6**), the lightest orange line is parallel to the *x*-axis at 3.5 of the *y*-axis. This reads as no matter how many alter ties exist, a network composed of eight Latinos always leads to an average outcome identification of 3.5 after 50 runs of the model with 50 time steps. In the purple graph below, all lines have a positive trend. So the more Latinos there are, no matter how many alter ties exist, the higher is the average Latino outcome identification after 50 simulation runs. In addition, alter ties seem to matter the most when there are five or six Latinos. In the standard deviation plot of the negative effect model for Latino identification (upper left graph in **Table 7**), the orange line for six Latino alters approaches a v-shape form and can be read as, no matter how many alter ties exist, a network composed of six Latino alters compared to other network compositions has the widest spread of possible outcomes considering 50 simulation runs. Likewise, the purple graph below shows that no matter how many alter ties exist, the standard deviation of the mean is the highest for six Latino alters. In general, the higher the standard deviation of the mean is, the greater is also the spectrum of possible outcomes after 50 simulation runs or, differently said, the lower is the consistency of simulation outcomes. Next, we present a selection of our results by mechanism or effect.

First, the *positive effect* model (identical to the mixed effect model of α = 1) always led to complete Latino identification (= 6) when there were at least six Latino alters. Likewise, complete U.S. identification (= 6) was reached when there were at least six European-American alters (identical to a maximum of two Latino alters) (**Table 6**). Second, complete identification with both cultures was also reached, for instance, when there were three to five Latino alters in a completely connected network (= 28 alter ties). Third, having no alters from one ethnicity resulted in the corresponding identification to be stable at 3.5. Fourth, when the ratio between the two ethnic groups was 4:4, the maximum of both identifications was reached or almost reached (smallest identification value 5.816), no matter how many alter ties existed. Fifth, the more alters were connected to each other, the less alters of one ethnicity were needed to result in maximum identification with that ethnicity. Sixth, when having only one alter from one ethnicity, ties hardly mattered for the result of the corresponding outcome identification (value approximately between 4.1 and 4.2; standard deviation relatively stable across ties, **Table 7**). This is because there are no other same ethnicity alters that this alter could have ties with. So the influence of this alter is stable even when the total amount of alter ties in the network increases. In contrast, ties between alters mattered the most when there were two alters of one ethnicity. Then, identification with that particular culture increased with the increase in amount of alter ties. That is because with two alters of the same ethnicity there can be only up to one tie between them, which results in a degree centrality of one for both alters. The more overall alter ties there are, the higher the probability that there is a tie between these two alters. Seventh, the standard deviation of the mean was the greatest for two alters of that ethnicity, no matter how many ties existed, with reaching the maximum when there was a medium amount of alter ties, and reaching a lower value when there were either no, few, or many ties. However, the standard deviation varied the most for three alters of that ethnicity.

The *negative effect* model yielded a pattern of results similar to the ones above but mirrored. First, complete disidentification (= 1) with one culture was reached when at least six alters belonged to the other culture no matter how many ties existed (**Table 6**). Second, complete disidentification with both cultures was also reached, for instance, when there were between three and five Latino alters in a completely connected network. Third, the identification outcome with one culture was stable at 3.5 across number of alter ties when all alters belonged to that culture. Fourth, when the ratio of the two ethnic groups was 4:4, both identifications reached or almost reached their minimum, no matter how many alter ties were present. Fifth, the more connected to each other alters were, the less alters of one ethnicity were needed to result in minimum identification with the other ethnicity. Sixth, ties mattered the least for the change in identification with one culture when the network was composed of seven alters of that culture (e.g., Latino identification somewhat stable around 2.9 when seven Latinos were present, no matter how many ties). The amount of alter ties mattered the most for the change in cultural identification when there were six alters from the same culture (analogously to having two alters of the same culture in the positive effect). Seventh, the standard deviation of the mean across ties was the greatest for six alters of the traced culture, but varied the most across ties when five of these alters were present (**Table 7**).

Selected results for the *mixed effects* model are shown in **Tables 8**, **9**. When α is 1, results are the same as in the positive effect model, and when α is 0, results are the same as in the negative effect model. When α is smaller than 0.5, the positive effect is smaller than the negative effect. When α is greater than 0.5, the positive effect is bigger than the negative effect. Only when α is equal to 0.5, both effects are equally important in the model. The results of two alphas that complement each other to 1 are mirrored. For example, results for α equal to 0.4 mirror the results of α equal to 0.6.

**Table 8:** Means for exemplary mixed effects.

	Mixed effects		
	
	α = 0.2	α = 0.5	α = 0.8
Latino ID			
	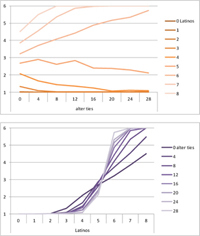	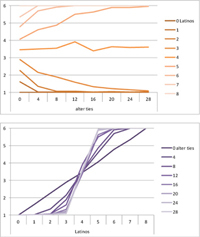	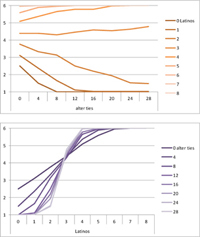

US ID			
	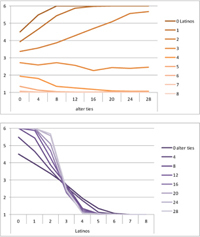	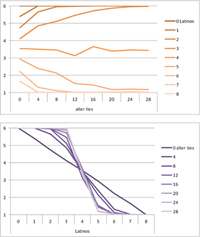	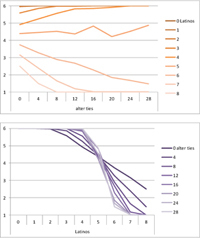


*Measurement points are shown as lines for visual simplicity. Some of the lines may be difficult to see as they overlap.*

**Table 9:** Standard deviations of the means for exemplary mixed effects.

	Mixed effects		
	
	α = 0.2	α = 0.5	α = 0.8
Latino ID			
	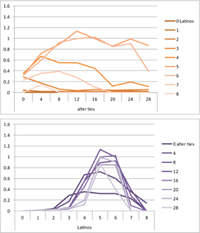	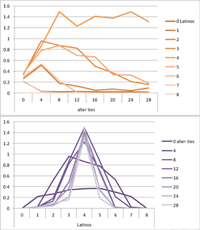	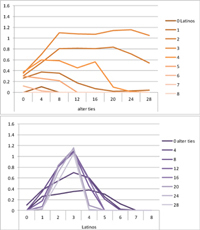

US ID			
	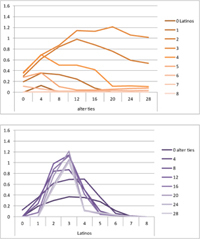	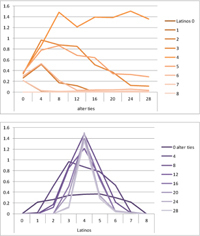	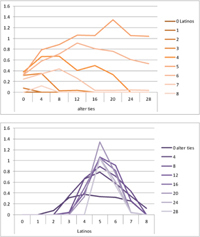


*Measurement points are shown as lines for visual simplicity. Some of the lines may be difficult to see as they overlap.*

In the special case where both effects were the same, ties did not or almost not matter for the outcome identification when there were no Latinos, four Latinos or eight Latinos (Latino identification was then 1, oscillating around 3.5 or 6, respectively; inverse for U.S. identification; **Table 8**). In contrast, ties had the most effect on the increase in cultural identification when there were five alters of that culture, and the most effect on the decrease in cultural identification when three alters of that culture were present. Further, the standard deviation of the mean was highest when alters from both cultures were equally present in the network, no matter how many ties existed (**Table 9**). Generally, the standard deviation of the mean decreased the more ties alters had.

For α equal to 0.2 (negative effect stronger than positive effect), having had five alters or less from one ethnicity, decreased the corresponding outcome identification in relation to the initial identification, while six alters or more led to an increase. Next, ties mattered the most in the case of six alters (**Table 8**), and the standard deviation of the mean was highest for five and six alters (**Table 9**). The results for α equal to 0.8 (positive effect stronger than negative effect) are the mirrored results of α equal to 0.2. For an α of 0.8, having had two alters or less from one ethnicity, decreased the corresponding outcome identification in relation to the initial identification of 3.5, while three alters or more led to an increase. Also, ties mattered the most in the case of two alters (**Table 8**), and the standard deviation of the mean was highest for two and three alters (**Table 9**).

### Discussion

To further explore the processes examined in Study 1, in this second study, we modeled the dynamics between social networks and cultural identity, and tested models involving coexisting cultural identities, conflicting cultural identities, and a mixture of the two based on the principle of influence in networks. Keeping the number of social interactions constant, we varied the ratio between Latino and European-American alters and the amount of their ties. The influence of the alters on cultural identification was stronger the more same ethnicity alters were connected to each other. We thus did not only model the cultural context of interaction (i.e., interaction with Latino or with European-American alter), but also its structural aspects (i.e., amount of same ethnicity ties that alter of interaction has), by giving importance to the social interactions among alters.

We showed that social network structure and content matter, but not in a homogenous, straightforward way, which might explain why these effects are difficult to detect in regression analyses involving cross-sectional data. Some network constellations may lead to very different results (expressed in a high standard deviation of the mean) depending on which alter ego interacts with; yet, some of these constellations may follow a similar trend. Other, but much fewer, constellations might even be stable in their outcomes. In certain cases, just one additional actor from one culture can make a big difference depending on the number of ties in the identity negotiation process.

This simulation enables us to identify network constellations that lead to complete disidentification with one or both cultures over time. Individuals who do not identify with their ethnic culture nor with their host culture may be of particular interest, as they might develop a sense of belonging to a third culture. While this new culture might be a more inclusive one (e.g., a global culture, a blended culture representing a unique combination of heritage and majority culture), it could also be a more extreme one (e.g., identification with a political or religious radical group). Hence, ABM could, among other things, contribute to the understanding of how acculturating individuals attain radical, extremist identifications (Verkuyten and Yildiz, 2007; Hogg and Adelman, 2013). Additionally, these simulations allow us to detect network constellations in which ego ends up with complete identification with both cultures. Which identity negotiation mechanism takes place may depend on various determinants such as the social, cultural, and political context, psychological characteristics of the individual (e.g., character traits such as dispositional openness and affiliative needs, level of BII) and the perceived or objective similarity of the two cultures.

## General Discussion

In the present research, we examined how key acculturation variables (i.e., ethnic and host cultural identifications, BII) relate to the composition and structure of Latino-American biculturals’ personal social networks. Drawing on the idea of complex social contagion from network theory and applying it to the negotiation process of multiple identities, we argue that immigrants and their descendants may adopt changes in their cultural identifications as a result of receiving repeatedly culture-relevant information from multiple network members representing different social roles. Relying on a community sample of Latino-Americans, in Study 1, we showed that the interconnection of same ethnicity alters across different relationship domains (i.e., friends and colleagues) predicts cultural identification, while the group size of these ethnicities does not. For these participants, the interconnection of Latino friends and colleagues is negatively associated with U.S.-American identification, and the interconnection of European-American friends and colleagues is negatively linked to Latino identification. This unexpected pattern suggests that, at least for our Latino-American sample, both cultural identifications are embedded in subtractive or even conflicting social network structures. Further, for this sample, time in the US is positively related to U.S.-American culture, but negatively to Latino culture, although this effect (indicative of a subtractive or a zero-sum pattern) is stronger for Latino-Americans who perceive tension between the two cultures (i.e., biculturals low on BII). This interactive pattern lends support to the idea that biculturals who experience low BII (e.g., “I feel caught between two cultures”) might manage this feeling by disidentifiying from one of the cultures over time.

While Study 1 was cross-sectional and could only show static interpersonal differences in cultural identification, Study 2 examined intrapersonal changes dynamically. In the latter study, we modeled the dynamics between social networks and cultural identification with both ethnic and host cultures over time, and tested models involving coexisting cultural identities, conflicting cultural identities and a mixture of the two based on the principle of influence. In doing so, we included the cultural context of interaction (i.e., interaction with Latino or with European-American alter) and its structural aspects (i.e., amount of same ethnicity ties that alter of interaction has). We showed that network structure and content matter, but not necessarily in a consistent or homogenous way. Still, we were able to identify network constellations that lead to complete identification or complete disidentification with one or both cultures over time. While certain network constellations may be beneficial for developing harmonious multicultural identities, others may lead to risky patterns of cultural disidentification and radicalization.

We would like to draw attention to some limitations of the current research. First, the data of Study 1 is cross-sectional, which does not allow for any causal inferences. Although we argued that the immigrant’s network influences ethnic and host cultural identifications, the reverse (selection) is also possible and likely. Individuals may choose certain people to be part of their network and determine how to connect them depending on their cultural identifications (Veenstra et al., 2013). In the future, longitudinal studies could explore in what way immigrants’ cultural identifications determine who becomes a network member and how these network members get connected (selection), and in what way the composition and the structure of the network influence immigrants’ identifications with ethnic and host cultures over time (influence). Also, longitudinal data would provide a sequence of at least two observations, which then could be used for designing a stochastic ABM to disentangle the intertwined relationship between selection and influence.

A second possible limitation is the way we measured the network in Study 1. We elicited the network by making respondents draw their networks. As soon as the network structure gets a little bit complicated, this task becomes tricky and people might be more likely to forget relationships between their network members. This is an issue that can be solved easily in future studies by using software especially developed for collecting egocentric network data, such as the program EgoNet, that automatically and separately asks the participants about each possible alter pair, thus facilitating an accurate reporting of all possible connections between alters.^4^

A third limitation concerns the environment of the data collection of Study 1. As the data were collected at a Cinco de Mayo street festival, our Latino community sample is likely to have been biased in favor of immigrants with a strong Latino identification. However, notice that we do not use this data to make empirical claims about Latino multiculturalism and Latino bicultural identity; we rather use the findings from Study 1 to develop a theoretical model in Study 2 that predicts intrapersonal change in cultural identification based on different identity negotiation mechanisms. Future studies could try to balance the cultural setting to also include individuals with lower ethnic identification.

Fourth, sample size, low significance of effects, and the quality and reliability of the scales used in Study 1 may be an issue. The modest size of our sample makes detecting reliable significant effects more difficult. Especially the network variables in Study 1, for which we claim effects on cultural identification for, are of low significance. Nonetheless, we argue that the effects of network composition and structure exist, but are rather hard to show in regression analysis as the process of influence is dynamic and not straightforward, as we illustrate in Study 2.^5^

Fifth, some of our implicit culture-related assumptions might be debatable. For instance, due to logistical and time constrains, we were only able to gather information on the ethnicity of all alters, but not on their cultural identifications. It could be interesting to also include alters’ actual cultural identifications as these may be different from their ethnicity. However, egos reported their perceived ethnicity of alters, which might be different from the real ethnicity and closer to observable aspects of the actual cultural identification.

A sixth limitation refers to the model assumptions in Study 2. In real life, not all social interactions are random. While some interactions may be, others may depend on the past. A new model could include the effect of the past on future interactions, for example, by (a) making an interaction with an alter of the same ethnicity as in the previous interaction more likely, or by (b) allowing the interaction to have more influence on identity change if the past interaction was with an alter of the same ethnicity as the alter of the new interaction. Further, actors are not constant over time. They may appear and disappear from a network as ties, too, may evolve and dissolve. Nevertheless, the structure of a network seems to be rather stable even when there is a high turnover in alters, because the way people structure their networks is also affected by their personality (Kalish and Robins, 2006). In addition, even when there is an exchange of actors, certain compositional measures (e.g., percentage of women) remain relatively stable (Lubbers et al., 2010). Finally, future ABMs based on our model should report the average degree centrality of alters by ethnicity and the number of homophilous ties.

Despite all these limitations, our research has also some key strengths. First, Study 1 relied on a community sample rather than a convenience sample (e.g., university students), as is often the case in cultural and social-psychological research. Second, the network approach grasps a real-life situation of intercultural contact in contrast to commonly used self-reports, which are highly dependent on the respondent’s self-awareness and are influenced by a variety of biases (e.g., social desirability, wishful thinking, lying about interactions); thereby, the social network approach is a less obstrusive and more implicit data collection mode that yields less danger of being actively manipulated by the respondent. In that sense, we combined individuals’ *thoughts* on acculturation (self-reported cultural identifications) and their acculturative *behavior* (network). Third, the transference of the theoretical idea of complex social contagion to the negotiation process of multiple cultural identifications is novel. Fourth, in Study 2 we show that individuals’ cultural identifications are not only influenced by their contacts, but also by the interactions that these contacts have between each other. Using an ABM approach, we were able to show that a distinct pattern of social relations (i.e., network composition and structure) does not lead to one deterministic identity outcome. Instead, in most of the cases, many different outcomes are probable, although they might follow a similar trend. Only in rare cases the exact outcome can be foreseen. The fact, that network composition and structure may affect multiple cultural identifications in many different ways, and not necessarily in a homogenous manner, might explain why it is difficult to detect network effects in regression analyses involving cross-sectional data. Fifth, experimental research has shown that, in laboratory settings, depending on the available social cues, different cultural frames become salient, and that cultural identification can shift accordingly. Our research does not only contextualize the effects of these social cues in a real-life environment, but also includes structural aspects of it.

Apart from the suggestions already mentioned, future studies could explore other immigrant populations and receiving contexts (e.g., Asian-Americans in the US, Turkish immigrants in Germany), as well as other types of biculturals (e.g., refugees, indigenous, or colonized individuals). Moreover, the boundaries of the network could be defined more openly and include additional relationship domains, such as religious, political, and community enclaves. A mixed-methods research design including network visualizations may allow the respondent to change from being observed to being the observer, and, thus, permit the addition of interpretative information on the network’s content, structure and changes over time (Molina et al., 2014).

To conclude, this research contributes to the multiple identities literature, and theory on biculturalism and cultural identity negotiation more specifically, as well as the literature on egocentric social networks, by exploring the links between key acculturation variables (i.e., ethnic and host cultural identifications, BII) and the composition and structure of Latinos’ personal social networks in an U.S.-American context. First, our results indicate that the social networks of Latino-American biculturals are related to these individuals’ levels of cultural identifications, and that this link is not necessarily based on the composition of the network (e.g., number of Latinos or Americans in the network), as some previous research has shown, but rather on its structure (the interconnection of same ethnicity individuals across different relational domains). Second, this research illuminates the link between degree of exposure to the dominant U.S. culture and ethnic and host cultural identifications by showing that the temporal pattern of a stronger U.S. identification and a weaker Latino identification with the pass of time is particularly prominent among Latinos who perceive their cultural identities as incompatible (i.e., those lower in BII). This finding furthers our understanding of BII and solidifies its validity as a construct to understand how individuals perceive and negotiate multiple cultural involvements over time. Overall, the findings from our two social network studies speak to issues relevant to the integration of immigrants and other cultural minorities, and might be informative in developing intercultural policies and programs that foster both, cohesive social communities and harmonious multicultural identities. In an increasingly multicultural world, this involves the successful inclusion of individuals of different cultural backgrounds into individuals’ social networks, and the active prevention of risky patterns of identity disidentification or radicalization (Simon et al., 2013; Lyons-Padilla et al., 2015).

## Author Contributions

VB-M contributed to the design of the first study and collected the data. LR contributed to the design of the second study. She performed the data analysis and interpretation of both studies under the supervision of VB-M. LR drafted the manuscript, and VB-M provided critical revisions.

## Conflict of Interest Statement

The authors declare that the research was conducted in the absence of any commercial or financial relationships that could be construed as a potential conflict of interest.

## Footnotes

1 From now on, we will refer to the second group in shorthand as ‘colleagues’ to avoid repetition.

2 Zumbo et al. (2007) recommend using ordinal coefficient alpha when estimating the reliability based on Likert response items (i.e., binary and ordinal response scales). Ordinal alpha and Cronbach’s alpha are conceptually the same with the difference that ordinal alpha is derived from a polychoric correlation matrix and not from Pearson’s covariance matrix. We followed the instructions of Gadermann et al. (2012) for calculating ordinal alpha in the statistical software package R (R Development Core Team, 2011).

3 In an ABM, a state variable usually describes an attribute or a property of an agent (e.g., age, sex, size, ethnicity).

4 EgoNet is a free software developed by Chris McCarty and Martin Smith which can be downloaded at https://sourceforge.net/projects/egonet/.

5 In addition, when constructing response scales for items in the questionnaire, response options directly tapping into different strengths of identification levels should be preferred to agree/disagree scales (as in the case of cultural identification) (Saris et al., 2010).
